# Signalling mechanisms regulating phenotypic changes in breast cancer cells

**DOI:** 10.1042/BSR20140172

**Published:** 2015-03-18

**Authors:** Natalia Volinsky, Cormac J. McCarthy, Alex von Kriegsheim, Nina Saban, Mariko Okada-Hatakeyama, Walter Kolch, Boris N. Kholodenko

**Affiliations:** *Systems Biology Ireland, University College Dublin, Belfield, Dublin 4, Republic of Ireland; †Laboratory for Integrated Cellular Systems, RIKEN Center for Integrative Medical Sciences (IMS), Tsurumi-ku, Yokohama, Kanagawa, Japan; ‡Conway Institute of Biomolecular & Biomedical Research, University College Dublin, Belfield, Dublin 4, Republic of Ireland

**Keywords:** breast cancer, cell fate decisions, lipid accumulation, MCF-7, mTOR, PI3 kinase, receptor tyrosine kinases signalling pathways, ACLY, ATP citrate lyase, ACSL1, long-chain-fatty-acid–CoA ligase 1, CCD, charge-coupled device, CHK, Csk homology kinase, CSK, C-terminal Src kinase, DMEM, Dulbecco's modified Eagle's medium, EGF, epidermal growth factor, ERK, extracellular-signal-regulated kinase, HRG, heregulin, MAPK, mitogen-activated protein kinase, MEK, MAPK kinase, mTOR, mammalian target of rapamycin, mTORC, mTOR complex, NRG, neuregulin, PI3K, phosphoinositide 3 kinase, PIP2, phosphatidylinositol 4,5-bisphosphate, PIP3, phosphatidylinositol 3,4,5-bisphosphate, PPAR, peroxisome proliferator-activated receptor, PTEN, phosphatase and tensin homologue, RSK, ribosomal S6 kinase, RTK, receptor tyrosine kinase, SFK, Src family kinase, TCA, tricarboxylic acid

## Abstract

In MCF-7 breast cancer cells epidermal growth factor (EGF) induces cell proliferation, whereas heregulin (HRG)/neuregulin (NRG) induces irreversible phenotypic changes accompanied by lipid accumulation. Although these changes in breast cancer cells resemble processes that take place in the tissue, there is no understanding of signalling mechanisms regulating it. To identify molecular mechanisms mediating this cell-fate decision process, we applied different perturbations to pathways activated by these growth factors. The results demonstrate that phosphoinositide 3 (PI3) kinase (PI3K) and mammalian target of rapamycin (mTOR) complex (mTORC)1 activation is necessary for lipid accumulation that can also be induced by insulin, whereas stimulation of the extracellular-signal-regulated kinase (ERK) pathway is surprisingly dispensable. Interestingly, insulin exposure, as short as 4 h, was sufficient for triggering the lipid accumulation, whereas much longer treatment with HRG was required for achieving similar cellular response. Further, activation patterns of ATP citrate lyase (ACLY), an enzyme playing a central role in linking glycolytic and lipogenic pathways, suggest that lipids accumulated within cells are produced *de novo* rather than absorbed from the environment. In the present study, we demonstrate that PI3K pathway regulates phenotypic changes in breast cancer cells, whereas signal intensity and duration is crucial for cell fate decisions and commitment. Our findings reveal that MCF-7 cell fate decisions are controlled by a network of positive and negative regulators of both signalling and metabolic pathways.

## Abstract 2

Excessive production and accumulation of lipids is often observed in breast cancer tissue. In the current study, we investigate signalling mechanisms regulating this process using a model cell line.

## INTRODUCTION

Cell fate decisions drive key biological processes, such as proliferation, differentiation, de-differentiation, epithelial–mesenchymal transition and many more. Cells undergo phenotypic changes not only during embryonic development, but also within adult tissues as a part of various physiological and pathological processes. Exploring these processes using *in vitro* systems allows us to obtain a better understanding of signalling mechanisms regulating permanent changes in cellular phenotype. Multiple studies have demonstrated that cell fate decisions are determined not only by activation of certain signalling pathways, but also depend on the spatiotemporal dynamics, including signal duration and strength. A well-known example are PC12 cells, where a transient activation of the mitogen-activated protein kinase (MAPK)/extracellular-signal-regulated kinase (ERK) pathway induced by epidermal growth factor (EGF) leads to cell proliferation, whereas a sustained activation of the same pathway induced by nerve growth factor results in growth arrest and neuronal differentiation [1]. Similar phenomena, observed in a wide range of organisms, varying from yeast to mammals, underlie the temporal regulation of signalling pathways as a common mechanism in determining cell fate decisions [2,3].

The MCF-7 breast cancer cell line is a well-established *in vitro* model, where certain stimuli, such as heregulin (HRG)/neuregulin (NRG), can induce irreversible phenotypic changes that involve the massive accumulation of lipid droplets and were taken in previous publications as indicator of differentiation [4–6]. This phenomenon is observed also in several other breast cancer cell lines [7]. Multiple stimuli, including polyunsaturated fatty acids, docosahexaenoic acid (DHA) and eicosapentaenoic acid (EPA) [8], quinolines [9], peroxisome proliferator-activated receptor (PPAR)γ agonists [10] and retinoic acid [11], can induce lipid accumulation in MCF-7 cells. Lipid accumulation can also be achieved by physiologically relevant perturbations that modulate the activity or expression levels of ErbB receptors [5–7].

MCF-7 cells exhibit different responses depending on which ErbB receptor ligand they are stimulated with. EGF binds to the ErbB1 receptor (also known as EGF receptor), whereas HRG preferentially binds to ErbB3 and ErbB4. HRG stimulation leads to a marked change in the cell phenotype, inducing lipid accumulation, whereas EGF fails to do so [6]. In both cases, homo- or hetero-dimerization of ErbB receptors take place, eventually leading to receptor transactivation [12–14]. Different receptor–ligand affinities and receptor-specific inactivation mechanisms can result in diverse cellular responses, mainly due to the different strengths and durations of the respective signalling activities [6,15]. The HRG-mediated signalling response is stronger and more sustained than the EGF response, which may explain why HRG, but not EGF, can stimulate lipid accumulation. However, it is currently unknown what signalling pathways are responsible for these different biological outcomes. Moreover, the origin of lipids accumulating in these cells has not been elucidated.

Activation of receptor tyrosine kinases (RTKs), including ErbB receptors, induces the recruitment of multiple scaffolds, kinases, GDP/GTP exchange factors and other signalling molecules to RTKs, forming multi-protein complexes responsible for transmitting ligand-induced signalling responses. Phosphoinositide 3-kinase (PI3K), recruited to the plasma membrane by RTKs or their complexes with adaptor proteins, phosphorylates phosphatidylinositol 4,5-bisphosphate (PIP2), producing a secondary messenger phosphatidylinositol 3,4,5-bisphosphate (PIP3). One of the major PIP3 effector proteins is Akt (also known as protein kinase B). Once recruited to the membrane via its pleckstrin homology (PH) domain, Akt is phosphorylated and activated by phosphoinositide-dependent kinase-1(PDK1) and mammalian target of rapamycin (mTOR) complex 2 (mTORC2) [16–18]. Among other effector proteins, Akt induces activation of mTORC1, a kinase complex regulating multiple cellular processes, such as protein synthesis, autophagy, transcription and metabolic pathways [19,20]. Like the ERK pathway, the PI3K–Akt–mTORC1 signalling pathway is also involved in the control of cell fate decisions. In regulatory cluster of differentiation CD4 T-cells, mTOR is required for cell differentiation into Th1 (T helper cells 1) and Th17 (T helper cells 17) lineages [21]. This signalling pathway also promotes the differentiation of mesenchymal stem cells into adipocytes [22] and depending on the experimental system used, mTORC1 can promote or inhibit osteoclasts differentiation [23].

In the present paper, we have explored signalling and metabolic pathways responsible for lipid accumulation and phenotypic changes in MCF-7 breast cancer cells in response to external cues. Although it was previously demonstrated that ErbB receptors can trigger MCF-7 cell fate decisions, no signalling pathways regulating this phenomenon were identified so far [6,7]. The present study identifies the PI3K–mTOR axis as a major signalling pathway that controls MCF-7 cells' phenotypic changes that include lipid synthesis.

## EXPERIMENTAL

### Tissue culture and transfections

The MCF-7 breast cancer cell line was maintained under standard conditions in Dulbecco's modified Eagle's medium (DMEM; Gibco) supplemented with 10% FBS, glutamine and penicillin/streptomycin. For siRNA transfections HiPerFect reagent (Qiagen) was used according to supplier's protocol. Briefly, 0.5×10^6^ cells in antibiotics-free media were seeded into each well of a standard 24-well plate. For each well, 6 μl of HiPerFect reagent and 8 pmole of siRNA were mixed with 100 μl of serum free DMEM media, incubated for 5–10 min and added to the cells. Eight hours later, when cells were fully attached, the transfection solution was replaced with starvation media, DMEM containing glutamine only. Cells were stimulated 20–24 h later. siRNA used: siGENOME SMARTpool–Human CSK (C-terminal Src kinase), ON-TARGET plus SMARTpool Human PTEN (phosphatase and tensin homologue), non-targeting ON-TARGET plus si CONTROL, all purchased from ThermoSientific/Dharmacon.

### Lipid accumulation assay

We slightly modified the previously published method [6] as follows: 0.4×10^6^ cells/well were seeded in a standard 24-well plate. Cells were starved 20–24 h prior to stimulation and treated with media containing 1% serum only or with addition of 10nM EGF from Roche, 2 nM recombinant human NRG1-β1/HRG1-β1 EGF domain from R&D or 10 μg/ml insulin (Sigma–Aldrich), unless otherwise indicated. For serum-free lipid accumulation experiments, the stimuli were added to media containing glutamine and antibiotics only. Inhibitors were added to the media as indicated: PD184352, PP2 (Sigma–Aldrich), AZD-8055 (JS Research Chemicals Trading), LY294002, rapamycin, TBCA (tetrabromocinnamic acid) (EMD Millipore/Calbiochem). Media containing stimuli and/or inhibitors were changed after 2–3 days. Cells were grown in a constant presence of stimuli and/or inhibitors for 5 days, unless otherwise specified, and then fixed with 10% paraformaldehyde. Then cells were washed once with water, once with 60% isopropanol and stained with Oil Red O solution for 1 h followed by three washes with water. For cell staining, fresh solution was prepared; 0.35% w/v Oil Red O (Sigma–Aldrich) stock solution was mixed with water in 6:4 ratio and filtered through 0.22 μm filter 1 h later. Lipid accumulation was quantified by extracting Oil Red from stained cells with isopropanol and measuring light absorbance at 500 nm. Following extraction of the Oil Red dye cells were stained with 5% Giemsa solution. Plates were scanned with an Epson perfection V750 PRO scanner and Giemsa dye intensity in each well was quantified using the ImageJ software. Lipids accumulation was normalized by dividing the Oil Red O value by the Giemsa value. Then, an average value of untreated cells was subtracted from all the wells in an experiment to remove background cell staining. Values were further normalized by dividing them by an average value of HRG treated cells obtained in the same experiment.

### Western blotting

Cells were seeded at densities similar to lipid accumulation assay and serum starved for 16–20 h. Approximately 2 h before stimulation, media were replaced with fresh starvation media to remove growth factors that may have been secreted by starved cells. Where indicated inhibitors were added 30 min prior to stimulation. Cells were stimulated with 10 nM EGF, 10 nM HRG or 10 μg/ml insulin for the times indicated. Cells were washed with 1× ice-cold PBS and lysed in buffer containing 1% SDS, 150 mM NaCl, 10 mM Tris buffer, pH 7.5, protease inhibitors cocktail (P8340), phosphatase inhibitors cocktails 2 and 3 and benzonase nuclease, all from Sigma–Aldrich. Antibodies used: PTEN, ribosomal S6 kinase (RSK)1, Akt1 (Santa Cruz), phospho-ERK, ERK1/2 (Sigma–Aldrich), pAkt S473 (Abcam), pSrc Y416, Src, pATP cytrate lyase (pACLY) S455 and ACLY, (mammalian long-chain acyl-CoA synthetase) (Cell Signaling Technologies). Standard Western blotting procedure was followed by enhanced chemiluminescence (ECL) procedure; bands were detected by CCD (charge-coupled device) camera (Advanced Molecular Vision). All bands detected were within the CCD camera sensitivity range. Bands intensity was quantified with ImageJ software.

## RESULTS

### PI3K signalling pathway determines cell ability to accumulate lipids

To examine cellular processes regulating phenotypic changes, we treated MCF-7 cells with selective inhibitors affecting two major signalling pathways induced by ErbB receptors in this cell line, i.e. the ERK/MAPK and PI3K pathways.

**Figure 1:**
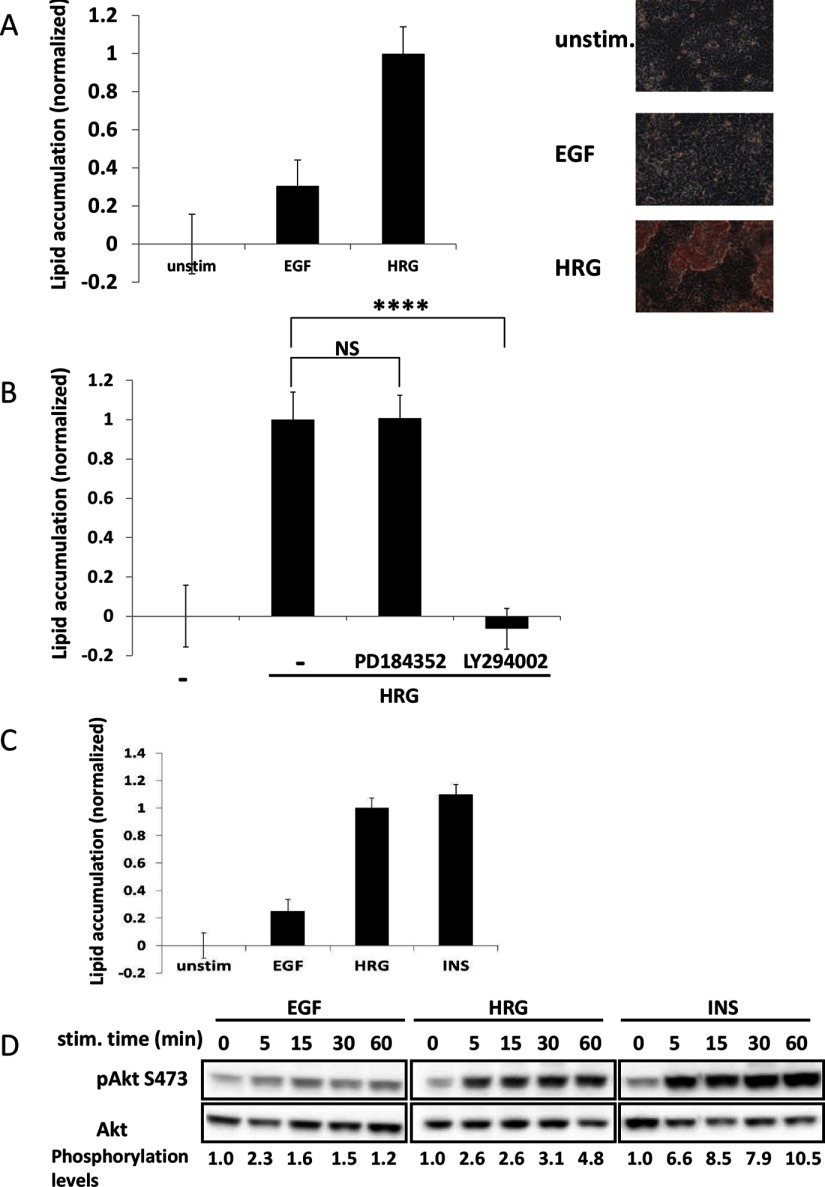
Cell differentiation depends on PI3K but not MAPK pathway (**A**) Starved cells were grown in control media (1% serum) or in media containing 10 nM EGF or 2 nM HRG. Five days later, cells were fixed and stained with Oil Red O dye to stain neutral lipids accumulated within the cells (right panel), followed by Giemsa staining. Oil Red values were divided by Giemsa values to normalize lipid accumulation to cell number. All values were normalized to HRG treated cells. Shown in the present study are values representing average of four independent experiments, each done in quadruplicate (*n*=16) ± S.D. (left panel). (**B**) Cells were grown in control media or media containing 2 nM HRG; 20 μM PD184352 (MEK inhibitor) or 10 μM LY294002 (PI3K inhibitor) were added to the media where indicated. Lipid accumulation was quantified as in (**A**). Data represent an average of three independent experiments, each preformed in quadruplicate (*n*=12) ± S.D. (**C**) Cells were treated as in (**A**) with addition of 10 μg/ml insulin treatment (*n*=12). (**D**) Starved cells were stimulated with 10 nM EGF, 2 nM HRG or 10 μg/ml insulin for times indicated. Cells were lysed and Akt S473 phosphorylation was detected by standard Western blotting. Total Akt was used as loading control. Akt S473 phosphorylation levels were calculated by dividing densitometry values of pAkt by total Akt values and normalizing to untreated cells. Shown in the present study is the best representative of four independent experiments.

First, we verified previous findings [6] that HRG, but not EGF, induces lipid accumulation in MCF-7 cells (Figures 1A). Both EGF and HRG activate the MAPK/ERK signalling cascade; however, with different kinetics [6]. To test whether lipid accumulation is regulated by ERK activation, we stimulated MCF-7 cells with HRG in the presence of a specific MEK (MAPK kinase) inhibitor, PD184352. To our surprise, this inhibitor did not have any significant effect on lipid accumulation (Figure 1B), despite efficient inhibition of MEK kinase activity, as validated by measuring the ERK phosphorylation status (Supplementary Figure S1A). Since HRG induces stronger and more sustained PI3K activation (as measured by Akt activation) compared with EGF (Figure 1D; [6]), we hypothesized that PI3K might determine the ability of MCF-7 cells to undergo phenotypic changes. Indeed, treatment with the PI3K inhibitor, LY294002, completely prevented HRG-induced lipid accumulation (Figure 1B). Based on this finding, we hypothesized that other stimuli capable of inducing sustained PI3K activation also should promote lipid accumulation. Indeed, insulin, which is a strong activator of PI3K but a weak activator of ERK signalling, induced lipid accumulation comparable with HRG-induced phenotypic changes (Figures 1C and 1D).

Cross-talk between signalling pathways induced by different stimuli is a common phenomenon that can be mediated by multiple mechanisms [24–26]. For instance, cell co-stimulation with insulin and low levels of EGF, both being weak activators of ERK, induces synergic ERK activation in human embryonic kidney (HEK)293 cells [27]. Since experiments with MCF-7 cells were performed in the presence of 1% serum, we had to exclude the possibility that the observed phenotypic changes arise from synergic ERK activation by insulin and low doses of growth factors present in serum. Therefore, we performed lipid accumulation experiments in serum-free media. Although the complete lack of serum decreased stimuli-induced lipid accumulation by approximately half, compared with media containing 1% serum, the response to insulin was still comparable to the response observed with HRG stimulation (Supplementary Figure S1B). These data demonstrate that insulin induces lipid accumulation by itself, rather than through potential synergism with growth factors and cytokines present in the serum.

**Figure 2:**
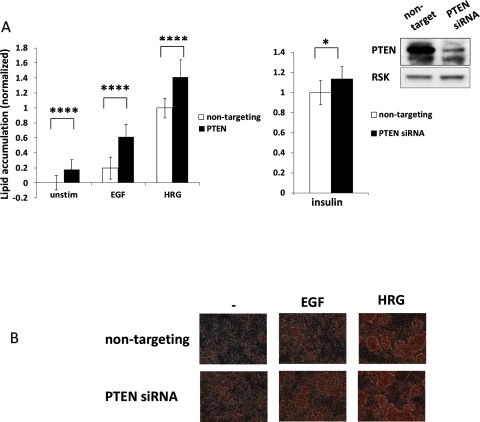
PTEN down-regulation promotes lipid accumulation (**A**) Cells were transfected with scrambled or PTEN targeting siRNA. Starved cells were grown in control media or in the presence of EGF or HRG (left panel, *n*=28) or insulin (middle panel, *n*=12) ± S.D. Lipid accumulation was quantified as in (**A**). To assess siRNA mediated knockdown, cells were lysed 6 days after transfection and PTEN expression levels were assessed by standard Western blotting. RSK protein was used as loading control (right panel). (**B**) Shown in the present study are representative images of cells from (**A**), stained with Oil Red O. Statistical significance was calculated by a standard *t*test, *P<0.05, **P<0.01, ***P<0.001, ****P<0.0001.

Another way to increase intracellular PIP3 levels is to inhibit negative regulator(s) of PIP3 formation. The lipid and protein phosphatase PTEN catalyses PIP3 de-phosphorylation into PIP2. By directly antagonizing PI3K activity, PTEN is one of the major negative regulators of the PI3K pathway [28,29]. To test whether lipid accumulation would be increased by PTEN down-regulation, cells were transfected with PTEN targeting siRNA followed by serum starvation and stimulation. PTEN down-regulation significantly increased lipid accumulation in cells stimulated with both HRG and EGF. Moreover, increased lipid accumulation was detected even in unstimulated cells, where under normal conditions there is no or very little lipid accumulation (Figure 2A, left panel and Figure 2B). Interestingly, PTEN knockdown had a much smaller effect in insulin treated cells (Figure 2A, middle panel), which is consistent with much stronger activation of the PI3K pathway induced by insulin compared with HRG (Figure 1D). Overall, these results clearly demonstrate that the phenotypic change is controlled by the PI3K pathway rather than by ERK pathway.

### mTORC1 controls MCF-7 cell phenotypic change

**Figure 3:**
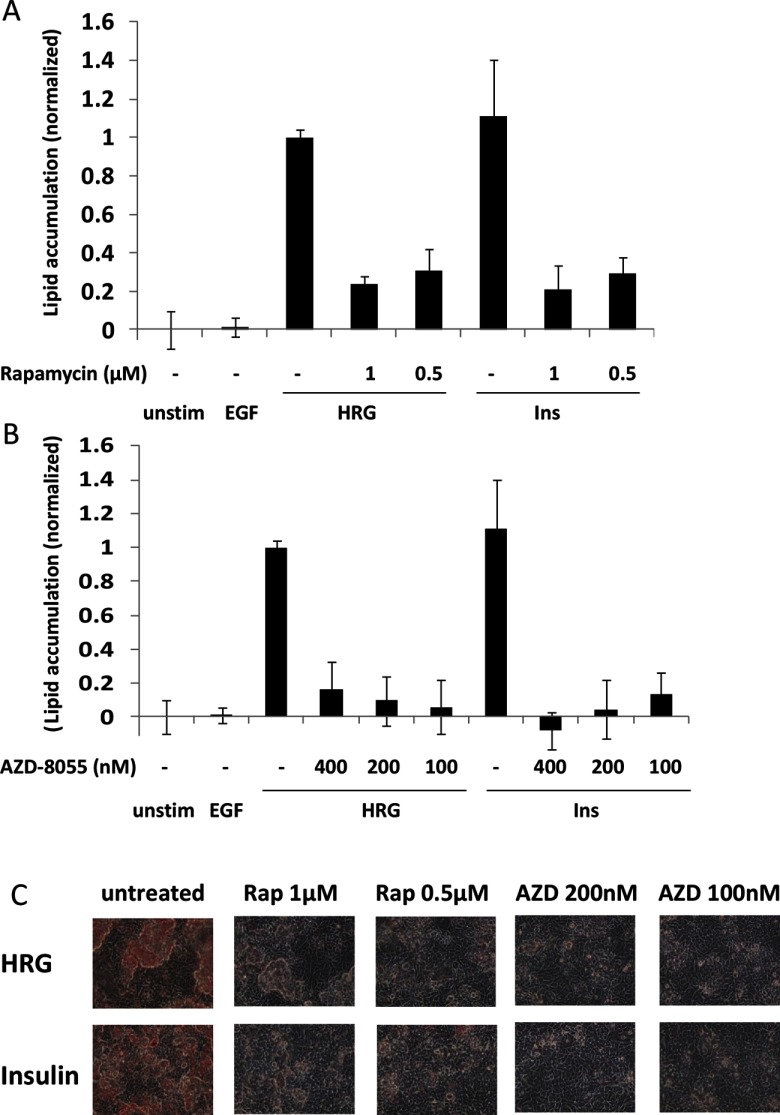
mTORC1 is required for cell differentiation (**A** and **B**) Starved cells were grown in control media (1% FBS) or in presence of 10 nM EGF, 2 nM HRG or 10 μg/ml insulin, as indicated. mTOR inhibitors, rapamycin (**A**) or AZD-8055 (**B**) were added to the media, where indicated. After 5 days, cells were fixed and lipid accumulation was quantified. Data shown are the best representative of five independent experiments done in quadruplicate ± S.D. (**C**) Shown are representative images of cells from (**A** and **B**), stained with Oil Red O.

PI3K activation and subsequent PIP3 production induce activation of multiple signalling molecules, including Akt, which is a main effector of PI3K. Therefore, we probed PI3K- and Akt-dependent pathways in more detail. An important Akt effector, especially for metabolic regulation, is the mTORC1. This multi-protein complex is activated by Akt, which inhibits the TSC1/2 (tuberous sclerosis 1/2) GTPase-activating protein (GAP) complex, thus leading to activation of the small G-protein Rheb (Ras homolog enriched in brain) and subsequent activation of its effector mTORC1 [30]. Rapamycin, a drug that inhibits mTORC1, substantially decreased lipid accumulation induced by both HRG and insulin (Figures 3A and 3C). To validate these results, we also used an ATP competitive mTOR kinase inhibitor, AZD-8055, which affects both mTORC1 and mTORC2 [31]. AZD-8055 suppressed lipid accumulation in response to HRG or insulin even stronger than rapamycin (Figures 3B and 3C). These data confirm that mTORC1 plays a major role in mediating MCF-7 phenotype and that additional contribution of mTORC2-dependent but mTORC1-independent molecules to lipid accumulation might take place.

### Src family kinases are required for lipid accumulation

**Figure 4:**
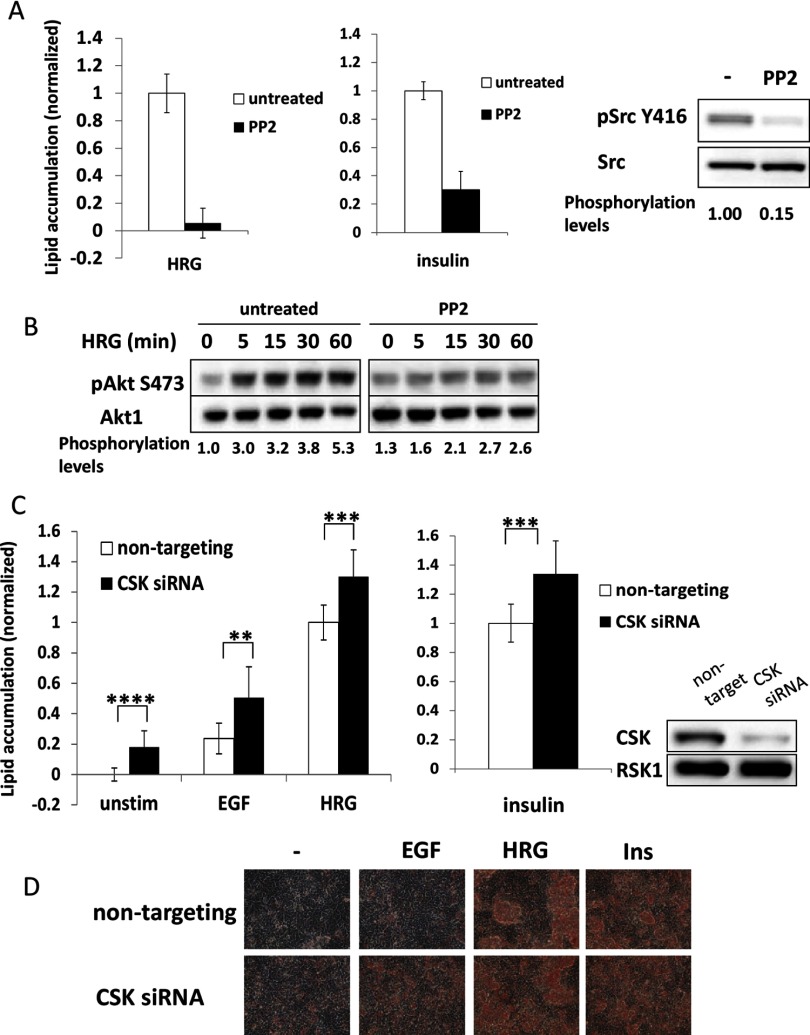
SFKs are required for MCF-7 phenotypic change (**A**) Starved cells were stimulated with HRG (left panel) or insulin (middle panel) in the presence of 10 μM PP2 (SFK inhibitor), where indicated. After 5 days, cells were fixed and lipids accumulated were quantified. Data presented are a representative of six independent experiments performed in quadruplicate. To confirm inhibitor efficiency, cells treated with PP2 or left untreated were lysed and Src phosphorylation on Tyr^416^ was detected by standard Western blotting (left panel). Src phosphorylation levels were calculated by dividing densitometry values of pSrc by total Src values and normalizing to untreated cells. (**B**) Starved cells were pretreated with 10 μM PP2 for 30 min, as indicated, followed by HRG stimulation. Cells were lysed and Akt Ser^473^ phosphorylation levels were detected by standard Western blotting. Akt1 expression levels were used as loading control. Akt Ser^473^phosphorylation levels were calculated by dividing densitometry values of pAkt by total Akt values and normalizing to untreated cells. Data presented are a representative of three independent experiments. (**C**) Cells were transfected with scrambled or CSK targeting siRNA. Starved cells were grown in control media or in a presence of EGF or HRG (left panel) or insulin (middle panel). Cells were fixed and lipid accumulation was quantified. Data presented are an average of three independent experiments each done in quadruplicate (*n*=12). To assess siRNA mediated knockdown efficiency, cells were lysed 6 days after transfection and CSK expression levels were measured by standard Western blotting. RSK protein was used as loading control (right panel). (**D**) Shown are representative images of cells from (**C**), stained with Oil Red O. Statistical significance was calculated by a standard *t*test, *P<0.05, **P<0.01, ***P<0.001, ****P<0.0001.

Having documented the central role of the PI3K and mTORC1 pathways in lipid accumulation, we sought to determine how RTKs activate the PI3K pathway. Src family kinases (SFKs) are regulated by the ErbB receptors, as well as by other RTKs, such as the insulin-like growth factor receptor [32–34]. To test whether SFKs are involved in MCF-7 phenotypic changes, cells were treated with the SFK-specific inhibitor PP2. PP2 potently reduced Src Y416 phosphorylation (Figure 4A, right panel). Src Y416 and equivalent sites in other SFKs, is an auto-phosphorylation site in the kinase activation loop, whose phosphorylation is necessary for full kinase activation [35] and hence is a direct readout of SFK catalytic activity [36]. Cells treated with PP2 failed to produce lipids in response to HRG or insulin (Figure 4A, left and middle panels). Consistent with this biological effect, PP2 also strongly inhibited the activation of the PI3K–Akt pathway by HRG (Figure 4B).

These results suggested that SFK activity is necessary to induce lipid accumulation. A complementary way to validate this role of SFKs is by enhancing SFK catalytic activity. Phosphorylation on Tyr^527^ keeps Src in an inactive, auto-inhibited conformation induced by the intramolecular interaction of this phosphotyrosine with the SH2 (Src homology 2) domain. Tyr^527^ is phosphorylated by the CSK or Csk homology kinase (CHK), which are negative regulators of SFK [36]. In MCF-7 cell line, both CHK and CSK are expressed at the mRNA levels, but only CSK catalytic activity is detectable [37]. To increase SFK activity, we down-regulated CSK gene expression by transfecting cells with the corresponding siRNA (Figure 4C, right panel). As expected, CSK down-regulation led to increased lipid accumulation in all conditions (Figures 4C and 4D). Taken together, these results indicate that SFKs are required for PI3K activation and phenotypic changes taking place in MCF-7 cells.

### Lipids accumulated are produced *de novo*

**Figure 5:**
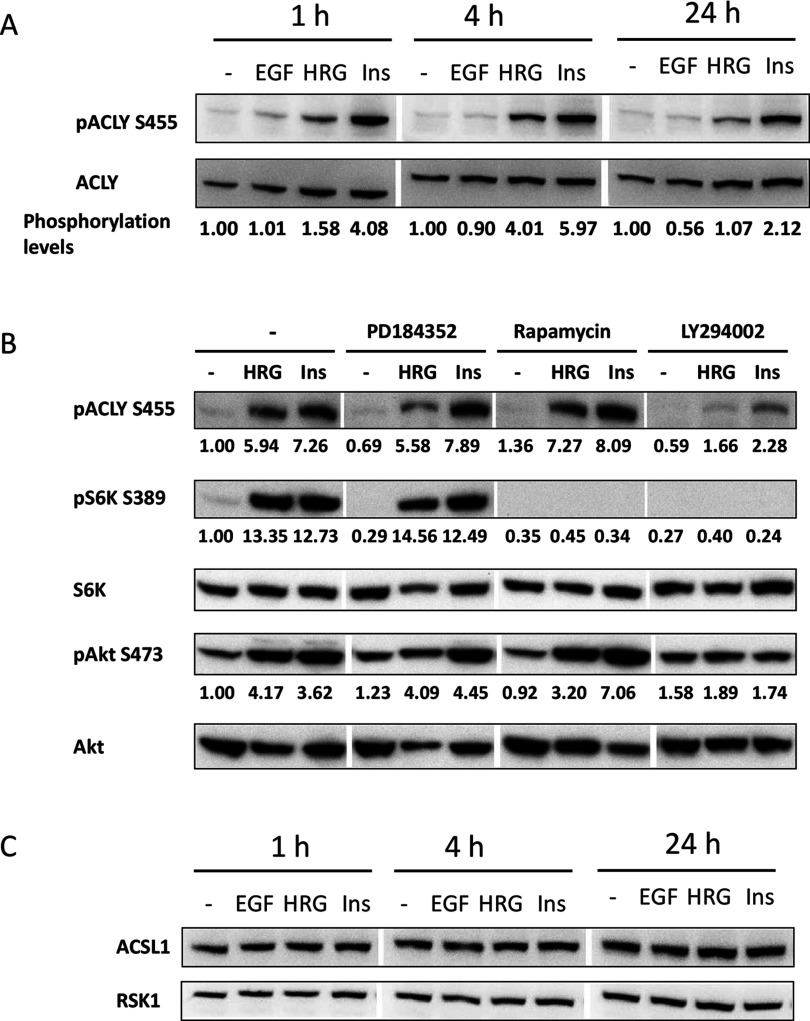
ACLY is activated in cells undergoing phenotypic changes (**A**) Starved cells were stimulated with 10 nM EGF, 2 nM HRG or 10 μg/ml insulin for times indicated. Cells were lysed and ACLY levels, total and phosphorylated at Ser^455^ were detected by standard Western blotting. Gaps between lanes indicate removal of irrelevant samples from the Western blot image. ACLY Ser^455^ phosphorylation levels were calculated by dividing densitometry values of the phosphorylated protein by densitometry values of total protein and normalized to untreated cells. Data shown are the best representative of four independent experiments. (**B**) Cells were treated with 2 nM HRG or 10 μg/ml insulin in a presence of 20 μM PD184352 (MEK inhibitor), 500 nM rapamycin (mTORC1 inhibitor) or 10 μM LY294002 (PI3K inhibitor), where indicated for 1 h. Cells were lysed and phosphorylation or expression levels of proteins were detected by a standard Western blotting, as indicated. ACLY Ser^455^ and S6K Ser^389^ phosphorylation levels were calculated by dividing densitometry values of the phosphorylated proteins by densitometry values of total S6K and normalized to untreated cells. Data shown are the best representative of three independent experiments. (**C**) Starved cells were stimulated with 10 nM EGF, 2 nM HRG or 10 μg/ml insulin for times indicated. Cells were lysed and ACSL1 expression levels were detected by standard Western blotting. RSK1 was used as loading control. Data shown are the best representative of three independent experiments.

There are two major mechanisms for acquiring fatty acids by mammalian cells. In an adult organism, most cells absorb lipids from the blood stream; whereas adipocytes and, in some cases, cancer cells synthesize lipids *de novo* [38]. In the latter case, carbohydrates, such as glucose, a main source of organic carbon, are required for fatty acids synthesis [39]. In this pathway, citrate produced from glucose within the tricarboxylic acid cycle (TCA cycle) is transported from the mitochondria into the cytoplasm where it is converted by ACLY into acetyl-CoA, an essential building block for fatty acid synthesis [38,39]. Therefore, we measured ACLY activity by assessing ACLY phosphorylation that correlates with its enzymatic activity [40,41]. Cell treatment with HRG or insulin resulted in increased ACLY phosphorylation, which lasted for at least 24 h. By contrast, EGF stimulation had a minimal impact on the ACLY phosphorylation levels (Figure 5A). To test what signalling pathways regulate ACLY activity, we treated cells with a panel of chemical inhibitors affecting major signalling molecules activated by HRG and insulin. MEK inhibitor, PD184352 did not affect ACLY phosphorylation. However, treatment with the PI3K inhibitor, LY294002, led to a substantial reduction in ACLY phosphorylation levels (Figure 5B). Interestingly, the PI3K-mediated ACLY phosphorylation was independent of mTORC1, as rapamycin had no effect on ACLY phosphorylation whereas efficiently abrogating the phosphorylation of p70 S6 kinase, a direct mTORC1 substrate. This is in agreement with previously published data demonstrating that Akt can directly phosphorylate ACLY and thus modulate its catalytic activity [42,43].

Utilization of fatty acids from the ‘environment’, whether their entrance into cells is mediated by transporting enzymes or is a passive penetration through lipophilic plasma membrane, requires the acyl-CoA synthetase family proteins [38]. These enzymes modify free fatty acids into acyl-CoA derivatives, thus retaining them in the cell and making them available for subsequent modifications and storage [44]. One of main enzymes in this family is long-chain-fatty-acid–CoA ligase 1 (ACSL1), which facilitates the absorption of fatty acids containing between 12 to 20 carbons. ACSL1 expression is regulated PPARs and changes in gene expression levels can be detected already after 6 h of stimulation with a PPAR agonist [45–49]. Moreover, its expression levels dramatically increase in the early stages of lactation [50]. MCF-7 cells treated with HRG or insulin up to 24 h did not exhibit any increase in ACSL1 protein expression levels (Figure 5C), indicating that HRG stimulation does not induce marked changes in the lipid transport from the media. Taken together, these data and the fact that cells are capable of producing and accumulating lipids even in a serum-free and therefore lipid-free media (Supplementary Figure 1B), suggest that lipids accumulated in stimulated MCF-7 breast cancer cells are produced *de novo* rather than absorbed from the media.

### The PI3K pathway promotes cell commitment

In the present study, we established that both HRG and insulin-induce lipid accumulation in MCF-7 cells and that this phenotypic change requires activation of the PI3K–mTOR signalling network. Moreover, despite the difference in the PI3K activation amplitude, these two stimuli induced lipids accumulation to a very similar extent (Figure 1C). In some biological processes, signal strength determines time required for cells to commit to a novel phenotype [51]. Based on this fact, we hypothesized that amplitude of PI3K pathway activation would determine time required for cells to commit to a phenotype induced.

**Figure 6:**
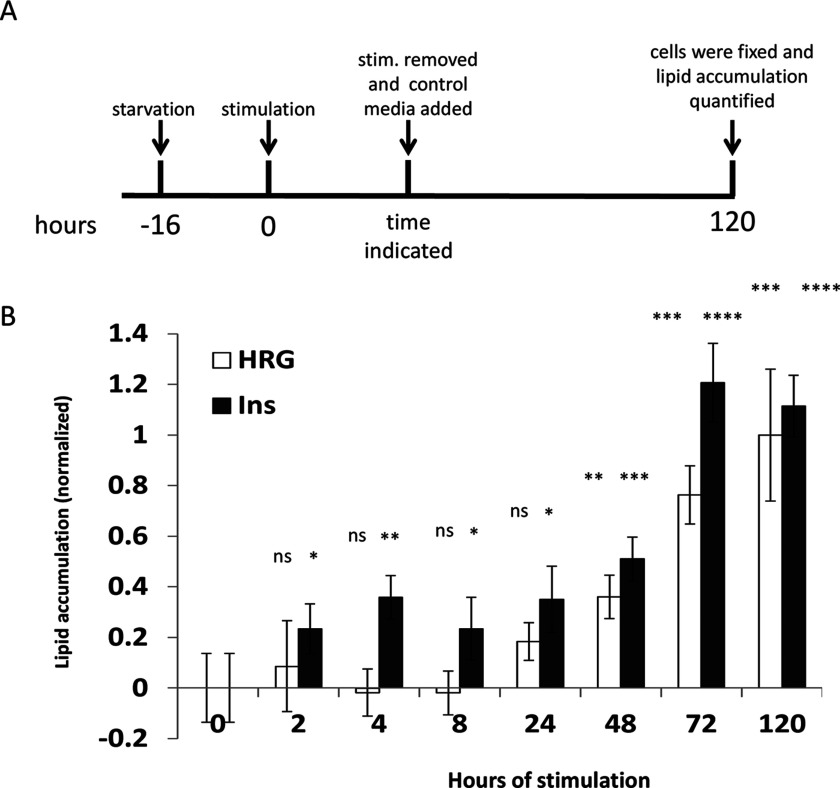
Cell commitment (**A**) A schematic presentation of the experiment performed. Cells were starved followed by treatment with HRG or insulin. At different time points varying between 2 and 72 h, stimulants were removed and cells were washed with a control media containing 1% serum only. Further, cells were grown in a control media up to 5 days from initial stimulation point (120 h), fixed and lipids accumulated were quantified as described in Figure 1(**A**). (**B**) Shown is the best representative experiment of three independent experiments performed in quadruplicates. For statistical analysis, all treatments were compared with cells constantly grown in media containing 1% serum only. Statistical significance was calculated by a standard *t*test, *P<0.05, **P<0.01, ***P<0.001, ****P<0.0001.

To identify when cells become committed to a novel phenotype and will continue to produce and accumulate lipids without external stimuli, we designed the following experiment. MCF-7 cells were stimulated with HRG or insulin for different time periods. Then, cells were washed and stimulant containing media was replaced with the control media containing 1% serum only. After that, lipid accumulation was monitored over 5 days, which is the time needed for fully acquiring the new phenotype (Figure 6A). Insulin induces accumulation of a low but statistically significant level of lipids even after exposures as short as 2–4 h, whereas 3-day stimulation was required to induce the maximal level of lipids accumulated. By contrast, very short HRG stimulation was inefficient in inducing lipid accumulation and only showed a significant phenotypic change at 2 days of HRG exposure, reaching its maximum after 5 days of continuous HRG stimulation (Figure 6B).

These data show that strong activation of the PI3K signalling pathway potentiates the ability of MCF-7 cells to commit to phenotypic changes induced by RTK ligands.

## DISCUSSION

Previous study demonstrated that HRG, but not EGF, can induce phenotypic changes in MCF-7 breast cancer cells [6]. However, the molecular mechanisms that control this biological process have never been elucidated. Our results demonstrate that phenotypic changes leading to accumulation of lipids are regulated by the PI3K–mTORC1 signalling axis, whereas ERK activity is dispensable.

An important feature of cells undergoing phenotypic changes is commitment, i.e. getting to a ‘point of no return’ where external cues are no longer required in order to fully acquire a new phenotype [52,53]. Several studies demonstrated that sustained compared with transient activation of signalling molecules can lead to different phenotypes [1–3]. Here we observe a similar phenomenon. The sustained activation of signalling pathways induced by HRG leads to phenotypic change in MCF-7 cells, whereas transient activation mediated by EGF fails to do so. Moreover, PI3K activation levels correlated with the time necessary for cells to commit to a novel phenotype and produce lipids even after a discontinuation of an extracellular signal. EGF hardly activates the PI3K pathway in MCF-7 cells, whereas HRG causes a sustained activation. Insulin induces sustained and even stronger pathway activation than HRG. These results suggest that the induction of phenotypic change requires the extended activation of PI3K and that the time to commitment is shortened with increasing amplitude of PI3K activation. Thus, a more prolonged HRG treatment is required for inducing lipid accumulation, whereas insulin treatment as short as 2–4 h is sufficient for triggering significant lipid production (Figure 6B). This phenomenon is similar to processes observed in other biological systems where cells commit to a new phenotype. For instance, in naive T-cells undergoing phenotypic changes upon interaction with antigen presenting cells, the signal strength and duration determine cell fate decisions [51]. Thus, whereas the initial cellular response to an external cue can develop within seconds or minutes, cell fate decisions are made over much longer periods of time which would often depends on the signal strength.

Observing MCF-7 breast cancer cells acquiring a novel phenotype that involves the massive accumulation of lipids, we asked how this phenomenon is regulated. Most tissues in an adult organism absorb fatty acids from the vascular system. The clear exception to that are adipocytes and cancer cells [38,39,54]. There is very limited information regarding the origin of lipids produced and secreted by the mammary gland. Some experimental data, including gene expression profiling, suggest that at least during the initial stages of lactation most lipids secreted into milk derive from the blood stream [50,55]. On the other hand, breast tumours often demonstrate hyper-activation of metabolic enzymes regulating *de novo* fatty acids synthesis, the phenomenon associated with Warburg effect [56–58]. The reason for cancer cell to undergo these metabolic changes is not clear yet and cannot be simply explained by hypoxia [39]. Nevertheless, this switch in cellular metabolism is observed in many types of cancer and is often associated with poor prognosis [43,59–63].

One of the major enzymes regulating absorbance of free fatty acids by cells is ACSL1. Expression of this enzyme is often triggered by external stimuli [47–49] and is greatly increased during the initial stages of lactation [50]. In the present study, we did not observe an increase in ACSL1 expression levels (Figure 5C). On the other hand, we found that lipid accumulation correlates with activation of ACLY, which is a central linker between glycolytic and lipogenic pathways [39,64,65]. ACLY is an enzyme converting citrate produced within TCA cycle and transported to the cytoplasm into acetyl-CoA, a metabolite required for fatty acids *de novo* synthesis as well as for other biological processes [40,64–66]. We found that PI3K pathway promotes ACLY activation and that mTORC1 is dispensable for this cellular response. This is in agreement with previously published studies where Akt was suggested as a kinase that phosphorylates and activates ACLY [41,43]. Several studies demonstrate an important role played by this enzyme in lung cancer development and suggest this enzyme as a potential target for cancer treatment [43,67–69]. Although an extreme abnormal activation of this enzyme in breast carcinoma samples was described already 35 years ago [58], ACLY contribution to breast cancer development and progression is yet unclear clear and requires further investigation.

**Figure 7:**
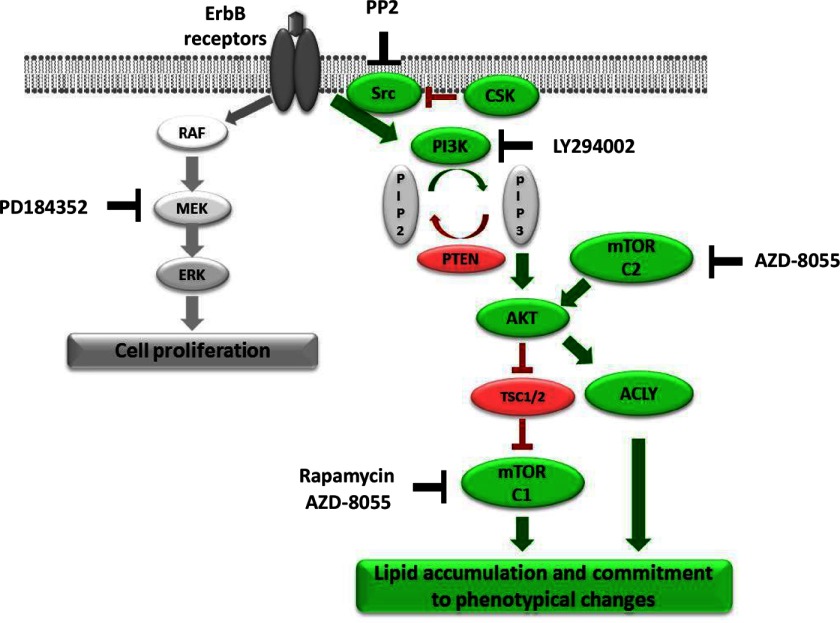
Signalling pathways regulating HRG-induced cell phenotypic changes and inhibitors used in the present study Upon HRG binding, ErbB3/4 receptors form hetero- or homo-dimers with other members of the ErbB family RTKs. Dimerization and transphosphorylation result in activation of two major signalling pathways: MAPK/ERK and PI3K. MAPK/ERK pathway activation promotes cell proliferation [70,71], whereas the PI3K pathway, often activated in breast cancer, was described as an anti-apoptotic and pro-survival pathway [72–77]. In the present study we demonstrate that the PI3K–mTOR axis also mediates phenotypic changes in MCF-7 breast cancer cells associated with massive production and accumulation of lipids. Akt activated downstream to the PI3K is a crucial element in mediating phenotypic changes; it activates ACLY that produces acetyl-CoA and mTORC1, crucial for lipids synthesis in these cells. Overall, ErbB receptors activation, leading to cell survival, proliferation and metabolic changes, contributes to tumour development and progression.

In summary, our results show that lipid accumulation in MCF-7 cells is a phenotypic cell fate decision that is mediated by the PI3K signalling pathway through a combined stimulation of endogenous lipid synthesis and the mTORC signalling pathway (Figure 7).

## Online data

Supplementary data

## References

[1] Marshall C.J. (1995). Specificity of receptor tyrosine kinase signaling: transient versus sustained extracellular signal-regulated kinase activation. Cell.

[2] Sabbagh W., Flatauer L.J., Bardwell A.J., Bardwell L. (2001). Specificity of MAP kinase signaling in yeast differentiation involves transient versus sustained MAPK activation. Mol. Cell.

[3] Singer A. (2002). New perspectives on a developmental dilemma: the kinetic signaling model and the importance of signal duration for the CD4/CD8 lineage decision. Curr. Opin. Immunol..

[4] Drabsch Y., Robert R.G., Gonda T.J. (2010). MYB suppresses differentiation and apoptosis of human breast cancer cells. Breast Cancer Res..

[5] Giani C., Casalini P., Pupa S.M., De Vecchi R., Ardini E., Colnaghi M.I., Giordano A., Menard S. (1998). Increased expression of c-erbB-2 in hormone-dependent breast cancer cells inhibits cell growth and induces differentiation. Oncogene.

[6] Nagashima T., Shimodaira H., Ide K., Nakakuki T., Tani Y., Takahashi K., Yumoto N., Hatakeyama M. (2007). Quantitative transcriptional control of ErbB receptor signaling undergoes graded to biphasic response for cell differentiation. J. Biol. Chem..

[7] Bacus S.S., Huberman E., Chin D., Kiguchi K., Simpson S., Lippman M., Lupu R. (1992). A ligand for the erbB-2 oncogene product (gp30) induces differentiation of human breast cancer cells. Cell Growth Differ..

[8] Chamras H., Ardashian A., Heber D., Glaspy J.A. (2002). Fatty acid modulation of MCF-7 human breast cancer cell proliferation, apoptosis and differentiation. J. Nutr. Biochem..

[9] Martirosyan A.R., Rahim-Bata R., Freeman A.B., Clarke C.D., Howard R.L., Strobl J.S. (2004). Differentiation-inducing quinolines as experimental breast cancer agents in the MCF-7 human breast cancer cell model. Biochem. Pharmacol..

[10] Kim K.Y., Kim S.S., Cheon H.G. (2006). Differential anti-proliferative actions of peroxisome proliferator-activated receptor-gamma agonists in MCF-7 breast cancer cells. Biochem. Pharmacol..

[11] Guilbaud N.F., Gas N., Dupont M.A., Valette A. (1990). Effects of differentiation-inducing agents on maturation of human MCF-7 breast cancer cells. J. Cell. Physiol..

[12] Earp H.S., Dawson T.L., Li X., Yu H. (1995). Heterodimerization and functional interaction between EGF receptor family members: a new signaling paradigm with implications for breast cancer research. Breast Cancer Res. Treat..

[13] Lemmon M.A., Schlessinger J. (2010). Cell signaling by receptor tyrosine kinases. Cell.

[14] Yarden Y., Sliwkowski M.X. (2001). Untangling the ErbB signalling network. Nat. Rev. Mol. Cell. Biol..

[15] Birtwistle M.R., Hatakeyama M., Yumoto N., Ogunnaike B.A., Hoek J.B., Kholodenko B.N. (2007). Ligand-dependent responses of the ErbB signaling network: experimental and modeling analyses. Mol. Syst. Biol..

[16] Bayascas J.R. (2008). Dissecting the role of the 3-phosphoinositide-dependent protein kinase-1 (PDK1) signalling pathways. Cell Cycle.

[17] Mora A., Komander D., van Aalten D.M., Alessi D.R. (2004). PDK1, the master regulator of AGC kinase signal transduction. Semin. Cell Dev. Biol..

[18] Sarbassov D.D., Guertin D.A., Ali S.M., Sabatini D.M. (2005). Phosphorylation and regulation of Akt/PKB by the rictor-mTOR complex. Science.

[19] Zoncu R., Efeyan A., Sabatini D.M. (2011). mTOR: from growth signal integration to cancer, diabetes and ageing. Nat. Rev. Mol. Cell. Biol..

[20] Dowling R.J., Topisirovic I., Fonseca B.D., Sonenberg N. (2010). Dissecting the role of mTOR: lessons from mTOR inhibitors. Biochim. Biophys. Acta.

[21] Heikamp E.B., Powell J.D. (2012). Sensing the immune microenvironment to coordinate T cell metabolism, differentiation & function. Semin. Immunol..

[22] Yu W., Chen Z., Zhang J., Zhang L., Ke H., Huang L., Peng Y., Zhang X., Li S., Lahn B.T., Xiang A.P. (2008). Critical role of phosphoinositide 3-kinase cascade in adipogenesis of human mesenchymal stem cells. Mol. Cell. Biochem..

[23] Xiang X., Zhao J., Xu G., Li Y., Zhang W. (2011). mTOR and the differentiation of mesenchymal stem cells. Acta Biochim. Biophys. Sin..

[24] Kolch W., Calder M., Gilbert D. (2005). When kinases meet mathematics: the systems biology of MAPK signalling. FEBS Lett..

[25] Volinsky N., Kholodenko B.N. (2013). Complexity of receptor tyrosine kinase signal processing. Cold Spring Harb. Perspect. Biol..

[26] Aksamitiene E., Kiyatkin A., Kholodenko B.N. (2012). Cross-talk between mitogenic Ras/MAPK and survival PI3K/Akt pathways: a fine balance. Biochem. Soc. Trans..

[27] Borisov N., Aksamitiene E., Kiyatkin A., Legewie S., Berkhout J., Maiwald T., Kaimachnikov N.P., Timmer J., Hoek J.B., Kholodenko B.N. (2009). Systems-level interactions between insulin-EGF networks amplify mitogenic signaling. Mol. Syst. Biol..

[28] Li L., Ross A.H. (2007). Why is PTEN an important tumor suppressor?. J. Cell. Biochem..

[29] Song M.S., Salmena L., Pandolfi P.P. (2012). The functions and regulation of the PTEN tumour suppressor. Nature reviews. Mol. Cell Biol..

[30] Huang J., Manning B.D. (2009). A complex interplay between Akt, TSC2 and the two mTOR complexes. Biochem. Soc. Trans..

[31] Chresta C.M., Davies B.R., Hickson I., Harding T., Cosulich S., Critchlow S.E., Vincent J.P., Ellston R., Jones D., Sini P. (2010). AZD8055 is a potent, selective, and orally bioavailable ATP-competitive mammalian target of rapamycin kinase inhibitor with in vitro and in vivo antitumor activity. Cancer Res..

[32] Ishizawar R.C., Miyake T., Parsons S.J. (2007). c-Src modulates ErbB2 and ErbB3 heterocomplex formation and function. Oncogene.

[33] Michels S., Trautmann M., Sievers E., Kindler D., Huss S., Renner M., Friedrichs N., Kirfel J., Steiner S., Endl E. (2013). SRC signaling is crucial in the growth of synovial sarcoma cells. Cancer Res..

[34] Finn R.S. (2008). Targeting Src in breast cancer. Ann. Oncol..

[35] Kmiecik T.E., Shalloway D. (1987). Activation and suppression of pp60c-src transforming ability by mutation of its primary sites of tyrosine phosphorylation. Cell.

[36] Roskoski R. (2005). Src kinase regulation by phosphorylation and dephosphorylation. Biochem. Biophys. Res. Commun..

[37] Bougeret C., Jiang S., Keydar I., Avraham H. (2001). Functional analysis of Csk and CHK kinases in breast cancer cells. J. Biol. Chem..

[38] Baumann J., Sevinsky C., Conklin D.S. (2013). Lipid biology of breast cancer. Biochim. Biophys. Acta.

[39] Vander Heiden M.G., Cantley L.C., Thompson C.B. (2009). Understanding the Warburg effect: the metabolic requirements of cell proliferation. Science.

[40] Zaidi N., Swinnen J.V., Smans K. (2012). ATP-citrate lyase: a key player in cancer metabolism. Cancer Res..

[41] Potapova I.A., El-Maghrabi M.R., Doronin S.V., Benjamin W.B. (2000). Phosphorylation of recombinant human ATP:citrate lyase by cAMP-dependent protein kinase abolishes homotropic allosteric regulation of the enzyme by citrate and increases the enzyme activity. Allosteric activation of ATP:citrate lyase by phosphorylated sugars. Biochemistry.

[42] Berwick D.C., Hers I., Heesom K.J., Moule S.K., Tavare J.M. (2002). The identification of ATP-citrate lyase as a protein kinase B (Akt) substrate in primary adipocytes. J. Biol. Chem..

[43] Migita T., Narita T., Nomura K., Miyagi E., Inazuka F., Matsuura M., Ushijima M., Mashima T., Seimiya H., Satoh Y. (2008). ATP citrate lyase: activation and therapeutic implications in non-small cell lung cancer. Cancer Res..

[44] Soupene E., Kuypers F.A. (2008). Mammalian long-chain acyl-CoA synthetases. Exp. Biol. Med..

[45] Schoonjans K., Staels B., Auwerx J. (1996). Role of the peroxisome proliferator-activated receptor (PPAR) in mediating the effects of fibrates and fatty acids on gene expression. J. Lipid Res..

[46] Rakhshandehroo M., Hooiveld G., Muller M., Kersten S. (2009). Comparative analysis of gene regulation by the transcription factor PPARalpha between mouse and human. PLoS One.

[47] Kanter J.E., Kramer F., Barnhart S., Averill M.M., Vivekanandan-Giri A., Vickery T., Li L.O., Becker L., Yuan W., Chait A. (2012). Diabetes promotes an inflammatory macrophage phenotype and atherosclerosis through acyl-CoA synthetase 1. Proc. Natl. Acad. Sci. U.S.A..

[48] Martin G., Schoonjans K., Lefebvre A.M., Staels B., Auwerx J. (1997). Coordinate regulation of the expression of the fatty acid transport protein and acyl-CoA synthetase genes by PPARalpha and PPARgamma activators. J. Biol. Chem..

[49] Rubinow K.B., Wall V.Z., Nelson J., Mar D., Bomsztyk K., Askari B., Lai M.A., Smith K.D., Han M.S., Vivekanandan-Giri A. (2013). Acyl-CoA synthetase 1 is induced by Gram-negative bacteria and lipopolysaccharide and is required for phospholipid turnover in stimulated macrophages. J. Biol. Chem..

[50] Bionaz M., Loor J.J. (2008). Gene networks driving bovine milk fat synthesis during the lactation cycle. BMC Genomics.

[51] Lanzavecchia A., Sallusto F. (2001). Antigen decoding by T lymphocytes: from synapses to fate determination. Nat. Immunol..

[52] Nagano M.C., Yeh J.R. (2013). The identity and fate decision control of spermatogonial stem cells: where is the point of no return?. Curr. Top. Dev. Biol..

[53] Welinder E., Ahsberg J., Sigvardsson M. (2011). B-lymphocyte commitment: identifying the point of no return. Semin. Immunol..

[54] Menendez J.A., Lupu R. (2007). Fatty acid synthase and the lipogenic phenotype in cancer pathogenesis. Nat. Rev. Cancer.

[55] McManaman J.L. (2014). Lipid transport in the lactating mammary gland. J. Mammary Gland Biol. Neoplasia.

[56] Menendez J.A., Vellon L., Mehmi I., Oza B.P., Ropero S., Colomer R., Lupu R. (2004). Inhibition of fatty acid synthase (FAS) suppresses HER2/neu (erbB-2) oncogene overexpression in cancer cells. Proc. Natl. Acad. Sci. U.S.A..

[57] Milgraum L.Z., Witters L.A., Pasternack G.R., Kuhajda F.P. (1997). Enzymes of the fatty acid synthesis pathway are highly expressed in in situ breast carcinoma. Clin. Cancer Res..

[58] Szutowicz A., Kwiatkowski J., Angielski S. (1979). Lipogenetic and glycolytic enzyme activities in carcinoma and nonmalignant diseases of the human breast. Br. J. Cancer..

[59] Furuta E., Okuda H., Kobayashi A., Watabe K. (2010). Metabolic genes in cancer: their roles in tumor progression and clinical implications. Biochim. Biophys. Acta.

[60] Kuhajda F.P., Jenner K., Wood F.D., Hennigar R.A., Jacobs L.B., Dick J.D., Pasternack G.R. (1994). Fatty acid synthesis: a potential selective target for antineoplastic therapy. Proc. Natl. Acad. Sci. U.S.A..

[61] Kuhajda F.P., Piantadosi S., Pasternack G.R. (1989). Haptoglobin-related protein (Hpr) epitopes in breast cancer as a predictor of recurrence of the disease. N. Engl. J. Med..

[62] Pitroda S.P., Khodarev N.N., Beckett M.A., Kufe D.W., Weichselbaum R.R. (2009). MUC1-induced alterations in a lipid metabolic gene network predict response of human breast cancers to tamoxifen treatment. Proc. Natl. Acad. Sci. U.S.A..

[63] Zaytseva Y.Y., Rychahou P.G., Gulhati P., Elliott V.A., Mustain W.C., O'Connor K., Morris A.J., Sunkara M., Weiss H.L., Lee E.Y., Evers B.M. (2012). Inhibition of fatty acid synthase attenuates CD44-associated signaling and reduces metastasis in colorectal cancer. Cancer Res..

[64] Bauer D.E., Hatzivassiliou G., Zhao F., Andreadis C., Thompson C.B. (2005). ATP citrate lyase is an important component of cell growth and transformation. Oncogene.

[65] Chypre M., Zaidi N., Smans K. (2012). ATP-citrate lyase: a mini-review. Biochem. Biophys. Res. Commun..

[66] Wellen K.E., Hatzivassiliou G., Sachdeva U.M., Bui T.V., Cross J.R., Thompson C.B. (2009). ATP-citrate lyase links cellular metabolism to histone acetylation. Science.

[67] Hanai J., Doro N., Sasaki A.T., Kobayashi S., Cantley L.C., Seth P., Sukhatme V.P. (2012). Inhibition of lung cancer growth: ATP citrate lyase knockdown and statin treatment leads to dual blockade of mitogen-activated protein kinase (MAPK) and phosphatidylinositol-3-kinase (PI3K)/AKT pathways. J. Cell. Physiol..

[68] Hanai J.I., Doro N., Seth P., Sukhatme V.P. (2013). ATP citrate lyase knockdown impacts cancer stem cells in vitro. Cell Death Dis..

[69] Lin R., Tao R., Gao X., Li T., Zhou X., Guan K.L., Xiong Y., Lei Q.Y. (2013). Acetylation stabilizes ATP-citrate lyase to promote lipid biosynthesis and tumor growth. Mol. Cell.

[70] Boldt S., Weidle U.H., Kolch W. (2002). The role of MAPK pathways in the action of chemotherapeutic drugs. Carcinogenesis.

[71] Karam M., Legay C., Auclair C., Ricort J.M. (2012). Protein kinase D1 stimulates proliferation and enhances tumorigenesis of MCF-7 human breast cancer cells through a MEK/ERK-dependent signaling pathway. Exp. Cell Res..

[72] Martini M., De Santis M.C., Braccini L., Gulluni F., Hirsch E. (2014). PI3K/AKT signaling pathway and cancer: an updated review. Ann. Med..

[73] Cairns R.A., Harris I.S., Mak T.W. (2011). Regulation of cancer cell metabolism. Nat. Rev. Cancer.

[74] Dillon R.L., White D.E., Muller W.J. (2007). The phosphatidyl inositol 3-kinase signaling network: implications for human breast cancer. Oncogene.

[75] Albert J.M., Kim K.W., Cao C., Lu B. (2006). Targeting the Akt/mammalian target of rapamycin pathway for radiosensitization of breast cancer. Mol. Cancer Ther..

[76] Vandermoere F., El Yazidi-Belkoura I., Adriaenssens E., Lemoine J., Hondermarck H. (2005). The antiapoptotic effect of fibroblast growth factor-2 is mediated through nuclear factor-kappaB activation induced via interaction between Akt and IkappaB kinase-beta in breast cancer cells. Oncogene.

[77] Gibson S., Tu S., Oyer R., Anderson S.M., Johnson G.L. (1999). Epidermal growth factor protects epithelial cells against Fas-induced apoptosis. Requirement for Akt activation. J. Biol. Chem..

