# Supplementary figures and images for: Signalling mechanisms regulating phenotypic changes in breast cancer cells
Source: Biosci Rep. 2015 Mar 18;35(2):e00178. doi: 10.1042/BSR20140172 (PMC4370098; doi:10.1042/BSR20140172)

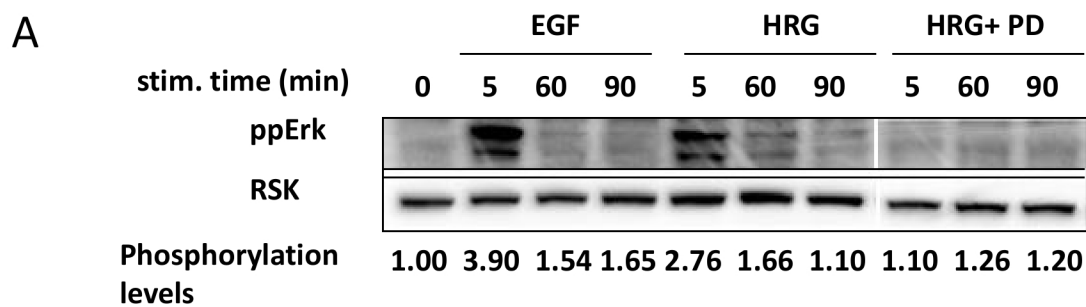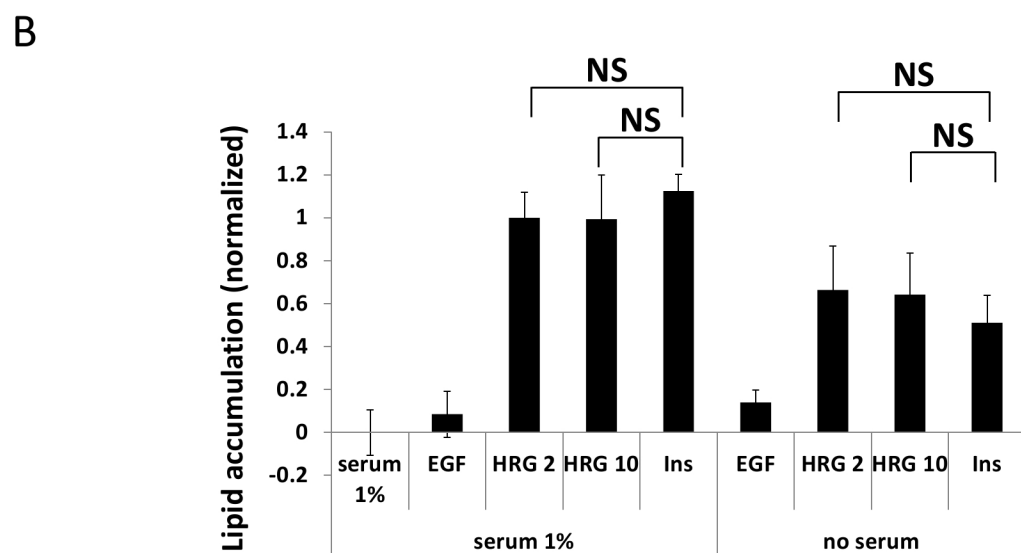

Supplementary Figure 1.

Supplement: Supplementary data [file bsr035e178ntsadd.pdf]
